# The effect of possible mediators on the association between chewing khat during pregnancy and fetal growth and newborn size at birth in Eastern Ethiopia

**DOI:** 10.1186/s12884-024-06243-2

**Published:** 2024-01-13

**Authors:** Amsalu Taye Wondemagegn, Miressa Bekana, Yonas Bekuretsion, Mekbeb Afework

**Affiliations:** 1 Department of Anatomy, School of Medicine, College of Health Sciences, Addis Ababa University, Addis Ababa, Ethiopia; 2 Department of Biomedical Sciences, School of Medicine, Debre Markos University, Debre Markos, Ethiopia; 3 Department of Obstetrics and Gynecology, School of Medicine, College of Health Sciences, Haramaya University, Harar, Ethiopia; 4 Department of Pathology, School of Medicine, College of Health Sciences, Addis Ababa University, Addis Ababa, Ethiopia

**Keywords:** Khat chewing, Substance use/misuse/abuse, Fetal growth, Fetal development, Small for gestational age, Ethiopia

## Abstract

**Introduction:**

Restriction in the growth of the fetus is a leading cause of stillbirth, neonatal mortality, and short- and long-term morbidity. Documented existing scientific evidence have shown the effects of maternal drugs use, alcohol drinking, tobacco smoking, cocaine use and heroin use on fetal growth restriction. However, data is lacking on the effects of khat chewing during pregnancy on fetal growth status and newborn size at birth. Therefore, the aim of the present study was to measure the effect of chewing khat during pregnancy on fetal growth and size at birth in eastern Ethiopia.

**Method:**

A cohort study was conducted in selected health institutions in eastern Ethiopia. All pregnant women fulfilled the eligibility criteria in the selected health institutions was the source population. The calculated sample size of exposed and unexposed groups included in the study, in total, was 344. Data collection was performed prospectively by interviewers administered questionnaires, and anthropometric, clinical and ultrasound measurements. Data was analyzed using SPSS version 27 and STATA version 16 software. The survival analysis (cox proportional hazards model) and generalized linear model (GLM) for the binomial family analysis were performed to estimate the crude and adjusted relative risk and attributable risk (AR) with corresponding 95% CI of chewing khat on fetal growth restriction. The mediation effect has been examined through Generalized Structural Equation Modeling (GSEM) analysis using the Stata ‘gsem’ command. Statistically significant association was declared at *p*-value less than 5%.

**Results:**

In the present study, the incidence of fetal growth restriction (FGR) among the study cohorts was 95 (29.7%); of this, 81 (85.3%) were among khat chewer cohorts. The relative risk of fetal growth restriction among khat chewer cohort mothers was significantly higher (aRR = 4.32; 95%CI 2.62–7.12). Moreover, the incidence of small for gestational age at birth among the present study cohorts was 100 (31.3%); 84 (84%) were from khat chewer cohorts’ deliveries. More importantly, in the present study, 98.95% of the ultrasound-identified fetuses with FGR were found to be SGA at birth. Hence, in the current study, FGR was highly associated with SGA at birth. In additional analysis, the regression coefficient of khat chewing during pregnancy on fetal growth restriction has been decreased in size from path o, β = 0.43, p < 0.001 to path o’, β = 0.32, *p* < 0.001, after adjusting for gestational hypertension and maternal anemia.

**Conclusion:**

In sum, the present study showed khat chewing during pregnancy is not simply affected the mothers, but it also affected the unborn fetuses. Therefore, the health workers as well as the local community and religious leaders should give high emphasis on provision of health education regarding the damage of chewing khat by pregnant mothers, with especial focus of the effects on their fetuses.

## Introduction

The growth and development of the unborn child/fetus is a multifaceted course and is relay on the many factors. Restricted fetal growth is the manifestation of a fundamental problem preventing the growth and development ability of unborn child. Fetal growth restriction (FGR) is a debatable problem [1]. The advancement of imaging technology such as ultrasound machines, are enabling to identify and understand more about this fetal problem.

Previously the word FGR is allotted to neonates with a birth/estimated fetal weight and/or length below the 10th percentile for their gestational age and whose abdominal circumference is below the 2.5th percentile [2], and it is a pathologic condition.

Currently, further parameters such as placental factors, biochemical factors and biometrics such as skin fold thickness and other anthropometric parameters of neonates like head circumference, biparietal diameter and others have been additionally considered to assess this disorder [3, 4]. On the other hand, small for gestational age (SGA) newborns are declared based on birth weight and/or length below the 10th percentile or less than 2 standard deviations (SD) for gestational age [5], and it may not be a pathologic state. Because SGA takes into account of only size and sex at birth, data on intrauterine growth status are not possible to obtain and hence, SGA may contain normal babies having only statures problem but with no pathological growth restriction. It has been reported that, there may be newborns which are simply genetically small, but normal without having increased morbidity and mortality [6]. Hence, SGA may be seen in fetal growth restricted newborn or non-restricted newborn, and thus not synonymous but highly related. It has been reported that, the smaller the unborn fetus or neonate, the higher the chance of being growth restricted [7]. The diagnosis of FGR is usually made during the prenatal period through the use of ultrasound.

Worldwide, fetal growth restriction have been observed in 5–10% of all pregnancies [8, 9], however its rate of occurrence is higher which is about 16–34% of births in low socioeconomic settings [10]. A cross sectional study conducted in northern parts of Ethiopia reported 23.5% prevalence of fetal growth restriction and 19.7% prevalence of SGA [11]. This variations in the occurrence of fetal growth restrictions may depends on the population under study, the geographic location of the study participants, the measurements and the standard growth curves used as a reference to compare [12].

Globally, fetal growth restriction is a leading cause of stillbirth, neonatal mortality, and short- and long-term morbidity [13, 14]. As such, FGR is the second leading cause of perinatal morbidity and mortality, next to preterm births and is accountable for about 20–30% of stillborn neonates [8, 10]; It is also the commonest cause of premature births and birth asphyxia [8].

Fetal growth and wellbeing are reliant on genetic/fetal, placental, and maternal factors. A relatively higher occurrence of fetal growth restriction in low- and middle-income countries may be attributed to nonharmony act of the fetal, placental, and maternal factors [15], so as to fulfill the needs of the unborn child through supporting the physiologic fluctuations of the women. Existing scientific evidence have documented the effects of maternal drugs use, alcohol drinking, tobacco smoke, cocaine, and heroin use on fetal growth restriction [8, 16]. However, data is lacking on the effects of khat chewing during pregnancy on fetal growth status and newborn size at birth. In addition, data on fetal growth restriction and SGA among births in Ethiopia is highly limited and nil in the present study area where khat chewing is highly practiced, including pregnant mothers. More importantly, there exists no previous study in Ethiopia conducted in a prospective study approach aimed at revealing the effect of khat chewing during pregnancy on fetal growth and size among deliveries in Ethiopia. Hence, the aim of the present study was to measure the effect of chewing khat during pregnancy on fetal growth and size in eastern Ethiopia.

## Methods and materials

### Study design and setting

A prospective cohort study of pregnant women who chewed khat and not chewed khat was conducted from August to December 2022 in selected health institutions of Dire Dawa administration, Harari region and Jigjiga city administration, eastern Ethiopia. Participants at high risk of adverse birth outcomes like having known major chronic illness such as diabetes mellitus and cardiovascular diseases; and having previous history of congenital anomalies were excluded. Moreover, those pregnant mothers with multiple pregnancy were also excluded.

### Sample size and sampling procedures

Open Epi version 3 statistical package was used to calculate the sample size by using 28.6% proportion of low birth weight in khat chewer groups (exposed) and 9.8% in non-khat chewers (non-exposed) from previous local study [17] and based on the assumptions of 95%, 80% power and r 1:1. The final sample size after using design effect 2 and adding 10% for loss to follow up is calculated to be 344 (172 non exposed and 172 exposed). Dire Dawa administration, Harari regional state and Jigjiga city were purposively selected due to exposure of interest. Then, 4 hospitals; 2 from Harari regional state, one from Dire Dawa administration and one from Jigjiga were taken. Pregnant mothers being in the second trimester and early third trimester (24–28 weeks) of pregnancy who visited the selected hospitals for the 1st or 2nd time during the study period was included until the required sample size of exposed and unexposed groups are fulfilled. The pregnancy follow-up contact period/time was at antenatal care appointments.

### Data collection procedures

Socio-demographic characteristics, past obstetrics related characteristics, substance use related characteristics and personal factors data was collected using structured and semi-structured questionnaire at entry to the study. The questionnaire was first prepared in English and then translated to local languages to facilitate understanding and ensure consistency during administration. In addition, anthropometric and clinical measurements were performed at entry, follow up time and delivery (end of pregnancy) to collect the necessary data for the independent variables.

Measurement of the exposure variable (khat chewing during pregnancy) was performed through maternal self-report. All pregnant women to be included in the study was first assessed for khat use at the first or second prenatal visit with the use of validated questionnaire. The WHO also suggested for identification of substance use during pregnancy by interview at antenatal care visits [18].

Khat use during current pregnancy is defined as ever chewing of khat during current pregnancy for at least 4 days per week which lasts for at least 4 h per chewing day or chewing for at least 4 days per week of at least 50–75 g of khat leaves per chewing day. This is based on previous local study [19] which found chewer’s of khat spend on average 3.75 h while chewing khat and chewed more than 75 g of khat leaves on single session.

Information regarding alcohol use comprising frequency and number of consumptions were obtained using questionnaire. Alcohol content standards for each beverage (beer, wine) was estimated and added to fix the total exposure volume of absolute alcohol (in grams per week). It is defined in previous literature that one standard drink is nearly equal to 0.5 ounces (14 g) absolute alcohol [20, 21]. Therefore, alcohol exposure status of participants can be categorized as < 1.5 drinks/week, 1.5–3.5 drinks/week, > 3.5-7 drinks/week, and > 7 drinks/week [20]. Furthermore, participants can be categorized as low (< 1.5 drinks/week), moderate (1.5–3.5 drinks/week) and high (> 3.5 drinks/week) alcohol drinkers. Nonuser of alcohol was those who did not report the use of any alcohol type during the current pregnancy.

In the present study, fetal growth restriction (FGR) is identified using (1) ultrasound when the estimated fetal weight is below 10th percentile for gestational age [22]. The major ultrasound parameters considered for identification of FGR were head circumference to abdominal circumference ratio (HC/AC), femur length to abdominal circumference ratio (FL/AC), amniotic fluid volume, and placental grading. In addition, (2) birth weight, sex of the neonates and gestational age at birth were used to determine small for gestational age [5] among all births; since FGR and SGA are highly related [5]. Small for gestational age at birth was declared when birth weight is below 10th percentile of the sex specific birth weight for gestational age reference curve [5]. Gestational age was calculated in terms of weeks using maternal recall of last menstrual period (LMP). Moreover, symphysis-fundal height (SFH) measurement in centimeters was conducted to approve LMP-based estimation of gestational age. All the measurement procedures were completed in accordance with relevant guidelines and regulations.

### Data quality management

To maintain data quality, training was given for data collectors and supervisors. Data were collected by qualified health professionals working on the selected hospitals. Different literatures have been reviewed to properly designed data collection material. Completeness and consistency of data was checked by the strict supervision of the supervisors and principal investigator.

### Statistical analysis

Data analyses were performed by SPSS version 27 and STATA version 16 software. Descriptive statistics such as median, interquartile range (IQR), and mean and standard deviation (SD) for continuous data and frequency distribution for categorical data is used to summarize the characteristics of the cohorts. Characteristics differences between khat chewers and non-khat chewer participants were examined using chi-square test (Pearson, *P*-values tested two-sided). The generalized linear model for the binomial family analysis were performed to estimate the crude and adjusted relative risk and attributable risk (AR) with corresponding 95% CI of chewing khat during pregnancy on fetal growth restriction. Variables with a univariate *p* value less than or equal to 0.25 was used in the multivariable model to estimate the aRR of chewing khat during pregnancy on fetal growth restriction. The relative risk with 95% confidence interval and *p*-values was used to measure the strength of association and to declare statistically significant association. In multivariable analysis model chewing khat during pregnancy was considered a statistically significant associated variable with fetal growth restriction at *p*-value less than 5%. Further mediation analysis was performed for observing the mediation effects of the possible mediators (gestational hypertension and maternal anemia) between the exposure variable (i.e., khat chewing during pregnancy) and outcome variable (i.e., fetal growth restriction). The analysis was performed using the Stata ‘gsem’ command on the drop-down menu bar. In addition, direct, indirect and total effects of khat chewing on fetal growth restriction has been calculated using the Stata ‘nlcom’ command.

### Ethical considerations

Ethical approval was obtained from Institutional Review Board of College of Health Sciences, Addis Ababa University. Permission was also obtained from the concerned bodies of Dire Dawa administration, Harari region and Somalia region. Moreover, informed written consent was obtained from the study cohorts.

## Results

Three hundred forty-four study participants were enrolled (172 non-khat chewers and 172 khat chewers) at the start of the study. Out of them, 320 (164 non-khat chewers and 156 khat chewers) finished the follow up resulting in a loss to follow up rate of 7%. The reasons of loss to follow up in the present study were refusal to continue (7 enrolled respondents; 5 chewers and 2 non-chewers), moved to other places (3 enrolled respondents; 2 chewers and 1 non-chewer), death (3 enrolled respondents; 2 chewers and 1 non-chewer) and home delivery (11 enrolled respondents; 7 chewers and 4 non-chewers).

### Sociodemographic characteristics of the study cohorts

In the present study, the mean (SD) age of the cohort mothers was 26.29 ± 5.49 years (range 17–45 years), with the majority (38.1%) aged between 25 and 29 years old. Most of the study cohorts was ethnic Oromo, 147 (45.9%); Muslim religion followers, 216 (67.5%) and living in urban area, 176 (55%). Of the study cohorts, 99 (30.9%) had no formal education and 80 (25%) had primary education level while 93 (29.1%) and 90(28.1%) of them were merchant and farmer in occupation respectively. The great majority, 269 (84.1%) of the study cohorts was married. The median [inter quartile range (IQR)] monthly household income of the study cohorts was 4800.0 ± 3500.0 Ethiopian birr (range 1,000.0–15,000.0 Ethiopian birr).

### Khat chewing characteristics of chewer study cohorts

The mean (SD) duration of khat chewing for chewer cohorts in this study was 34.77 ± 15.37 months (range 12–60 months), with the higher duration of chewing, 76 (48.7%) for 12–24 months. More than half, 82 (52.6%) of chewer cohorts had a khat chewing frequency of greater or equal to 4 days per a week. The median [inter quartile range (IQR)] amount of khat consumed at a single khat chewing session was 90 ± 50 g. Fifty-eight (37.2%), 56 (35.9%) and 42 (26.9%) of chewer cohorts consumed 50–75, 76–100 and > 100 g of khat per single khat chewing session respectively. The mean (SD) duration of khat chewing in a single chewing session was 3.95 ± 0.69 h, with the duration for majority of chewer cohorts, 122 (78.2%) 3–4 h.

### Comparison of behavioral characteristics of study cohorts

In total, 43; 31 (72.1%) chewer and 12 (27.9%) non-khat chewer study cohorts were consumers of alcohol of any type during their current pregnancy. Of them, 27(8.44%), 13 (4.1%) and 9 (2.81%) consumed beer, wine, and locally prepared alcohol (Tela) respectively. In terms of amount, 6 (15%) of the cohorts consumed 16.5 g of alcohol in a week; 14 (35%) of the study cohorts consumed 27.5-33 g of alcohol in a week, and the remaining cohorts, which is 20 (50%) consumed 49.5–55 g of alcohol in a week. Out of the total, 18 (5.63%) of the cohorts were practiced smoking of tobacco products, and almost all (95%) of the study cohorts were consumed coffee (Table 1).

**Table 1:** Comparison of behavioral characteristics of khat chewer and non-chewer cohorts in eastern Ethiopia, 2022 (*N* = 320)

Characteristics	Khat chewing practices of cohorts		*p*-value
	Chewers, Frequency (%)	Nonchewers, Frequency (%)	
Alcohol intake in last 1 months of pregnancy			
Yes	31 (72.1%)	12 (27.9%)	< 0.001
No	125 (45.1%)	152 (54.9%)	
Beer intake in last 1 months of pregnancy			
Yes	21 (77.8%)	6 (22.2%)	0.002
No	135 (46.1%)	158 (53.9%)	
Amount of beer consumed (in bottle and gram equivalents) in a week			
1 bottle (16.5 g of alcohol)	5 (83.3%)	1 (16.7%)	0.755
2 bottles (33 g of alcohol)	7 (70%)	3 (30%)	
3 bottles (49.5 g of alcohol)	9 (81.8%)	2 (18.2%)	
Wine intake in last 1 months			
Yes	8 (61.5%)	5 (38.5%)	0.346
No	148 (48.2%)	159 (51.8%)	
Amount of wine consumed (in glass and gram equivalents) in a week			
1 glass (27.5 g of alcohol)	2 (50%)	2 (50%)	0.569
2 glasses (55 g of alcohol)	6 (66.7%)	3 (33.3%)	
Homemade alcohol drinks (Tela)			
Yes	6 (66.7%)	3 (33.3%)	0.275
No	150 (48.2%)	161 (51.8%)	
Overall levels of alcohol consumed in a week (converted to standard measures)			
< 1.5drinks (16.5 g of alcohol) (low)	5 (83.3%)	1 (16.7%)	0.641
1.5-3.5drinks (27.5–33 g of alcohol) (moderate)	9 (64.3%)	5 (35.7%)	
> 3.5drinks (49.5–55 g of alcohol) (high)	15 (75%)	5 (25%)	
Smoking of any tobacco products			
Yes	14 (77.8%)	4 (22.2%)	0.011
No	142 (47%)	160 (53%)	
Frequency of tobacco smoking			
Daily	4 (57.1%)	3 (42.9%)	0.093
More than one day per a week	10 (90.9%)	1 (9.1%)	
Coffee use of study cohorts			
Yes	151 (49.7%)	153 (50.3%)	0.151
No	5 (31.3%)	11 (68.8%)	

### Obstetric distribution patterns of study cohorts

The majority, 174 (54.4%) of the study cohorts were multigravida (having > = 3 pregnancies); of them, 106 (60.9%) were chewer cohorts, and the rest 68 (39.1%) were non-chewer cohorts. Of total, 148 (46.3%) of the study cohorts were multipara (having > = 2 children); of them, 87 (58.8%) were chewer cohorts and the remaining, 61 (41.2%) non-chewer cohorts. One hundred four (32.5%) of the study cohorts had a previous history of spontaneous abortion; of them, 84 (81%) were khat chewer cohorts. Fifty (15.6%) of the study cohorts had a previous history of still birth; with 26 (52%) chewer cohorts and 24 (48%) non-chewer cohorts. The majority, 264 (82.5%) of the study cohorts gestational age at time of enrollment to the study was 24–26 weeks and the majority, 240 (75%) of the study cohorts had the first time visit of the hospitals at time of enrollment to the study. Only 37 (11.6%) of the study cohorts had at least 4 ANC visits at the end of delivery; of this, the majority (87%) of them was non-khat chewer cohorts (Table 2).

**Table 2:** Distribution of obstetric characteristics of study cohorts by their khat chewing practices in eastern Ethiopia, 2022 (*N* = 320)

Obstetric characteristics	Khat chewing status of study cohorts		*p*-value
	Chewers, Frequency (%)	Non-chewers, Frequency (%)	
Gravida			
1 (primigravida)	24 (28.6%)	60 (71.4%)	< 0.001
2 (secundigravida)	26 (41.9%)	36 (58.1%)	
>=3 (multigravida)	106 (60.9%)	68 (39.1%)	
Para			
0 (nullipara)	42 (38.2%)	68 (61.8%)	0.003
1 (primipara)	27 (43.5%)	35 (56.5%)	
>=2(multipara)	87 (58.8%)	61 (41.2%)	
Spontaneous abortion history			
Yes	84 (80.8%)	20 (19.2%)	< 0.001
No	72 (33.3%)	144 (66.7%)	
Previous still birth history			
Yes	26 (52%)	24 (48%)	0.617
No	130 (48.1%)	140 (51.9%)	
Gestational age at time of enrollments			
24–26 weeks	125 (47.3%)	139 (52.7%)	0.276
27–28 weeks	31 (55.4%)	25 (44.6%)	
ANC visits at time of enrollments			
1st	126 (52.5%)	114 (47.5%)	0.02
2nd	30 (37.5%)	50 (62.5%)	
Number of ANC visits attended at the end of delivery			
< 4 ANC visits	151 (53.4%)	132 (46.6%)	< 0.001
>=4 ANC visits	5 (13.5%)	32 (86.5%)	
History of malaria			
Yes	5 (45.5%)	6 (54.5%)	0.824
No	151 (48.9%)	158 (51.1%)	

### Physical measurements and umbilical cord status of the study cohorts

The overall mean (SD) weight of study cohorts was 63.28 ± 9.19 kg; of this 150 (46.9%) of them weighed between 61 and 70 kg. The mean (SD) height of the study cohorts was 157.46 ± 13.34 cm; with the highest cohorts, 205 (64.1%) measured above 157 cm high. The overall mean (SD) BMI of the cohorts was 25.7 ± 3.9 kg/m^2^ (range 17.6–37.8 kg/m^2^); of this, 8 (2.5%) of them had BMI < 18.5 kg/m^2^ and 50 (15.6%) had BMI > = 30 kg/m^2^. The mean (SD) MUAC of the cohorts was 26.92 ± 3.49 cm; of this, 46 (14.4%) of them had less than 23 cm and 19 (5.9%) had > = 33 cm (Table 3).

Moreover, the umbilical cord of 253 (79.1%) study cohorts was normally twisted; and the magnitude of under and hyper coiled umbilical cords among the study cohorts was 41 (12.8%), and 26 (8.1%) respectively. In addition, true knot was identified in 43 (13.4%) umbilical cords of the study cohorts (Table 3).

**Table 3:** Comparison of bodily measurements and umbilical cord status of khat chewer and non-khat chewer study cohorts in eastern Ethiopia, 2022 (*N* = 320)

Measurements	Khat chewing characteristics of study cohorts		*p*-value
	Chewers, Frequency (%)	Nonchewers, Frequency (%)	
Weight (in kg)			
< 50 kg	8 (47.1%)	9 (52.9%)	< 0.001
50-60 kg	63 (57.3%)	47 (42.7%)	
61-70 kg	54 (36%)	96 (64%)	
> 70 kg	31 (72.1%)	12 (27.9%)	
Mean (SD) weight (in kg)	63.48 ± 10.67	63.08 ± 7.55	0.697
Height (in cm)			
< 145 cm	18 (48.6%)	19 (51.4%)	0.967
145-157 cm	39 (50%)	39 (50%)	
> 157 cm	99 (48.3%)	106 (51.7%)	
Mean (SD) height (in cm)	157.31 ± 13.92	157.59 ± 12.81	0.85
BMI (in kg/m^2^)			
< 18.5	4 (50%)	4 (50%)	< 0.001
18.5–24.9	82 (53.9%)	70 (46.1%)	
25-29.9	35 (31.8%)	75 (68.2%)	
>=30	35 (70%)	15 (30%)	
Mean (SD) BMI (in kg/m^2^)	25.86 ± 4.29	25.61 ± 3.39	0.559
MUAC (in cm)			
< 23 cm	22 (47.8%)	24 (52.2%)	0.013
23-27.9 cm	77 (55.4%)	62 (44.6%)	
28-32.9 cm	44 (37.9%)	72 (62.1%)	
>=33 cm	13 (68.4%)	6 (31.6%)	
Mean (SD) MUAC (in cm)	26.96 ± 3.77	26.81 ± 3.24	0.701
Twists of umbilical cord			
Under coiled	29 (70.7%)	12 (29.3%)	0.002
Normal	111 (43.9%)	142 (56.1%)	
Hyper twisted	16 (61.5%)	10 (38.5%)	
True knots identified in umbilical cords			
Yes	27 (62.8%)	16 (37.2%)	0.048
No	129 (46.6%)	148 (53.4%)	

### The incidence of fetal growth restriction and small for gestational age and their association with khat chewing

The incidence of fetal growth restriction among the study cohorts was 95 (29.7%); of this, 81 (85.3%) were among khat chewer cohorts, and the remaining 14 (14.7%) were among non-khat chewer cohorts. Moreover, the incidence of small for gestational age at birth among the present study cohorts was 100 (31.3%); 84 (84%) were from khat chewer cohorts’ deliveries. More importantly, in the present study, 98.95% of the ultrasound-identified fetuses with FGR were found to be SGA at birth. Hence, in the current study, FGR was highly associated with SGA at birth.

As explained in Table 4, the GLM for the binomial family analysis revealed that, the adjusted relative risk of fetal growth restriction among khat chewer cohort mothers was about 4 times higher (aRR = 4.32; 95%CI 2.62–7.12) (*p* < 0.001) compared to non-khat chewer cohorts. In addition, the attributable risk of fetal growth restriction due to khat chewing was 43.4% (95%CI 34.46–52.32). Lastly, area of residence, cigarette smoking and true knots in umbilical cord was found to be significant variables associated with fetal growth restriction.

In the same way, analysis of the present study revealed that, the adjusted relative risk of small for gestational age among khat chewer cohorts was almost 4 times higher (aRR = 3.89; 95%CI 2.38–6.38) (*p* < 0.001) compared to non-khat chewer cohorts. Moreover, the attributable risk of small for gestational age due to khat chewing was 44.1% (95%CI 35-53.1) (*p* < 0.001). A similar pattern of findings is observed in the analysis of SGA and other variables considered in the FGR analysis. Accordingly, area of residence, urban (aRR = 0.43; 95%CI 0.25–0.75), cigarette smoking (aRR = 2.26; 95%CI 1.14–4.48) and true knots (aRR = 2.26; 95%CI 1.05–4.88) were the variables which showed a significant association with SGA.

**Table 4:** Comparison of the effects of khat chewing during pregnancy on fetal growth restriction among study cohorts in eastern Ethiopia, 2022 (*N* = 320)

Variables	Fetal growth restriction		aRR (95%CI)	*p*-value
	Yes, No (%)	No, No (%)		
**Khat chewing status**				
Chewers	81 (85.3%)	75 (33.3%)	4.32 (2.62–7.12)	< 0.001
Non chewers	14 (14.7%)	150 (66.7%)	1	
**Age group of** **study participants**				
<=24 years	28 (29.5%)	94 (41.8%)	0.85 (0.42–1.73)	> 0.05
25–29 years	35 (36.8%)	87 (38.7%)	1.29 (0.67–2.5)	
30–34 years	14 (14.7%)	30 (13.3%)	1.55 (0.6–3.97)	
>=35 years	18 (18.95%)	14 (6.2%)	1	
**Participants area of residence**				
Urban	27 (28.4%)	149 (66.2%)	0.46 (0.26–0.81)	< 0.05
Rural	68 (71.6%)	76 (33.8%)	1	
**Participants educational status**				
No formal education	43 (45.3%)	56 (24.9%)	1.4 (0.57–3.52)	> 0.05
Primary education	24 (25.3%)	56 (24.9%)	1.98 (0.78–5.04)	
Secondary education	16 (16.8%)	57 (25.3%)	1.2 (0.49–3.09)	
Tertiary education	12 (12.6%)	56 (24.9%)	1	
**Participants Occupation**				
House wife/ Homemaker	15 (15.8%)	53 (23.6%)	0.93 (0.29–3.04)	> 0.05
Farmer	29 (30.5%)	61 (27.1%)	1.16 (0.41–3.26)	
Employee	10 (10.5%)	40 (17.8%)	0.35 (0.10–1.18)	
Merchant	35 (36.8%)	58 (25.8%)	1.35 (0.48–3.75)	
Daily laborer	6 (6.3%)	13 (5.8%)	1	
**Alcohol use during current pregnancy**				
Yes	20 (21.1%)	23 (10.2%)	1.72 (0.89–3.27)	> 0.05
No	75 (78.9%)	202 (89.8%)	1	
**Cigarette smoking during current pregnancy**				
Yes	14 (14.7%)	4 (1.8%)	2.14 (1.03–4.44)	< 0.05
No	81 (85.3%)	221 (98.2%)	1	
**Total ANC visits attended**				
< 4 ANC visits	88 (92.6%)	195 (86.7%)	0.85 (0.34–2.14)	> 0.05
>=4 ANC visits	7 (7.4%)	30 (13.3%)	1	
**MUAC (in cm)**				
< 23 cm	16 (16.8%)	30 (13.3%)	1.12 (0.34–3.74)	> 0.05
23-27.9 cm	53 (55.8%)	86 (38.2%)	1.19 (0.42–3.39)	
28-32.9 cm	17 (17.9%)	99 (44%)	0.77 (0.26–2.30)	
>=33 cm	9 (9.5%)	10 (4.4%)	1	
**BMI (in kg/m** ^**2**^ **)**				
< 18.5	4 (4.2%)	4 (1.8%)	1.43 (0.40–5.09)	> 0.05
18.5–24.9	31 (32.6%)	121 (53.8%)	0.83 (0.44–1.57)	
25-29.9	30 (31.6%)	80 (35.6%)	1.95 (0.97–3.92)	
>=30	30 (31.6%)	20 (8.9%)	1	
**True knots in umbilical cords**				
Yes	33 (34.7%)	10 (4.4%)	2.35 (1.08–5.12)	< 0.05
No	62 (65.3%)	215 (95.6%)	1	

aRR: adjusted relative risk

### Mediation analysis results

**Fig. 1:**
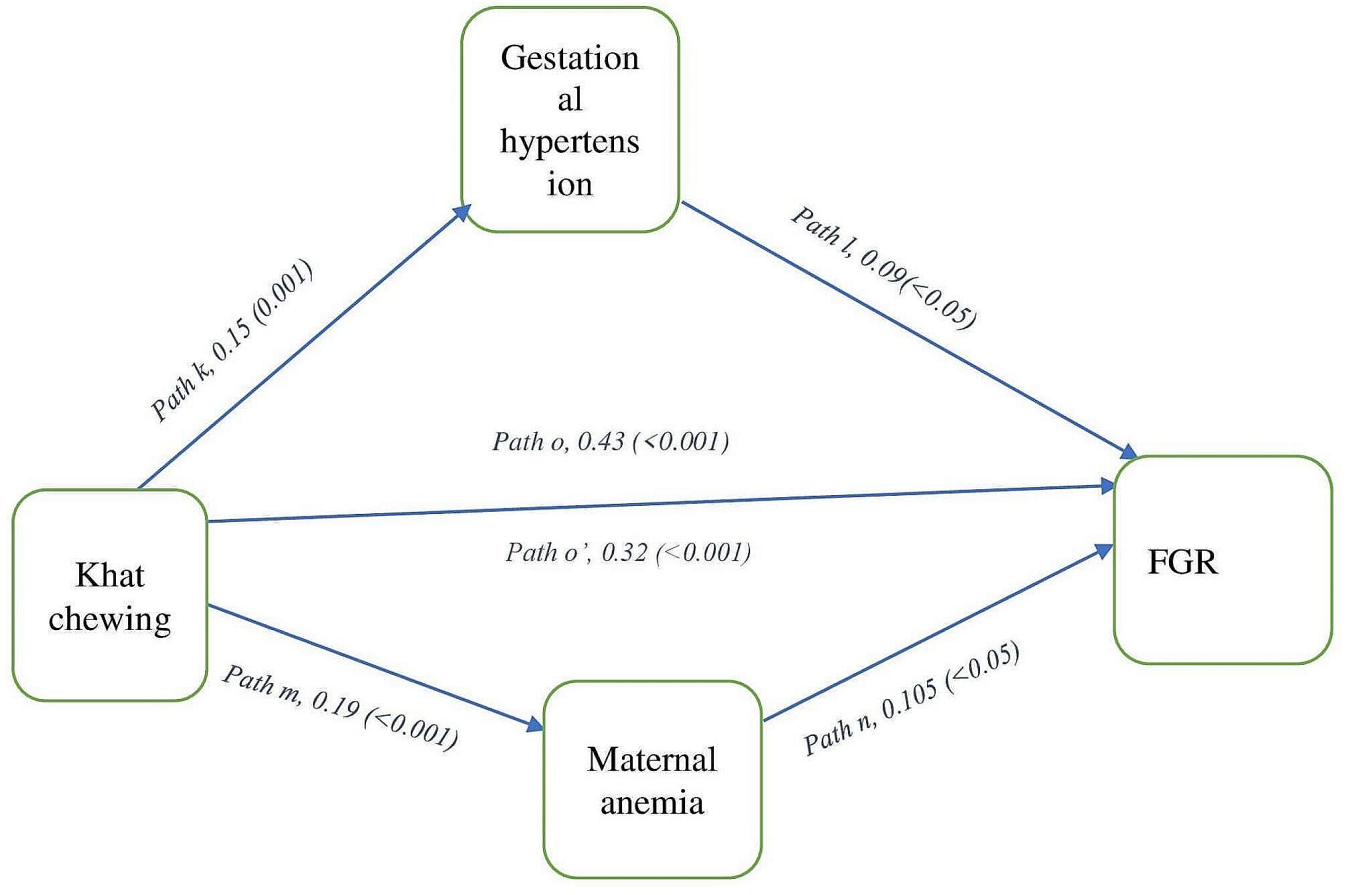
Showed the adjusted effect sizes of khat chewing during pregnancy on fetal growth restriction through the potential mediators. β(*p*-value) of path k, 1, m and n is the indirect effects of khat chewing during pregnancy on FGR through gestational hypertension and maternal anemia. β(*p*-value) of path o and o’ is the direct effects of khat chewing during pregnancy on FGR before and after adjusting for gestational hypertension and maternal anemia respectively

The mediation analysis results of the effect of khat chewing during pregnancy on fetal growth restriction is detailed in Tables 5 and Fig. 1. Khat chewing during pregnancy was significantly associated with FGR (path o, β = 0.43, p < 0.001). More importantly significant associations were also observed between khat chewing during gestation and gestational hypertension (path k, β = 0.15, p = 0.001), gestational hypertension and FGR (path l, β = 0.09, p < 0.05), khat chewing during gestation and maternal anemia (path m, β = 0.19, p < 0.001), maternal anemia and FGR (path n, β = 0.105, p < 0.05). After adjusting for gestational hypertension and maternal anemia, the regression coefficient of khat chewing during pregnancy has been decreased in size from path o, β = 0.43, p < 0.001 to path o’, β = 0.32, *p* < 0.001 (Fig. 1). Therefore, the present study revealed that the effect of khat chewing during pregnancy on fetal growth restriction was partially mediated by gestational hypertension and maternal anemia.

**Table 5:** The relationship between khat chewing during pregnancy, potential mediators and fetal growth restriction of the study cohorts in eastern Ethiopia, 2022 (*N* = 320): A generalized structural equation modeling analysis

Model		β*(95% CI)	*p*-value
Fetal growth restriction	Khat consumption effect on gestational hypertension	0.15 (0.06–0.24)	0.001
	Gestational hypertension effect on FGR	0.09 (0.011–0.11)	< 0.05
	Khat consumption effect on maternal anemia	0.19 (0.089–0.29)	< 0.001
	Maternal anemia effect on FGR	0.105 (0.016–0.19)	< 0.05
	Khat consumption effect on FGR before adjustment for gestational hypertension and maternal anemia	0.43 (0.35–0.52)	< 0.001
	Khat consumption effect on FGR after adjustment for gestational hypertension and maternal anemia	0.32 (0.24–0.43)	< 0.001

*=adjusted for maternal age, residence, education status, occupation status, alcohol use, tobacco smoke, ANC visits, MUAC, BMI, oligohydramnios, placental abruptio, true knots in umbilical cord

## Discussion

In the present follow-up study, the incidence of fetal growth restriction among the study cohorts was 29.7%. Moreover, the incidence of small for gestational age at birth among the present study cohorts was 31.3%. Although, the study populations and the study approaches are not comparable to discuss, a lower prevalence of FGR (23.5%) and SGA (19.7%) have been reported in a previous local study [11].

A significantly higher incidence and increased relative risk of fetal growth restriction was observed among khat chewer participants as compared to their non-khat chewer counterparts in the present follow up study. The probable explanations for this finding may be associated with extrauterine and intrauterine factors. The extrauterine environment may be a factor in the following ways. One, there may be differences in daily dietary intake of chewers and non-khat chewers. It was reported [23] that chewing khat may decrease the food appetite of pregnant mothers and hence, chewer pregnant mothers may consume less which may highly decreases the nutrient quantity needed for unborn fetus and then will affect its growth. In addition, since chewer pregnant mothers, even the poor, may give priority for buying khat, chewer pregnant mothers may be in lack of nutritious foods at household and then consume less food that may not satisfy the need of unborn fetus and as result will affect its growth [23]. In agreement with these elaborations’ additional mediation analysis in the present study found a significant association between khat chewing during pregnancy and maternal anemia and maternal anemia and fetal growth restriction. The other an increased relative risk of fetal growth restriction may be associated intra uterine environment such as placental and umbilical cord abnormalities. An experimental animal study has reported a decrease in placental blood flow due to vasoconstriction in the uteroplacental vessels among khat fed animals as compared to controls [24] and then this may lead to fetal growth restriction. This may be because the active constituent of khat, principally cathinone, an amphetamine like substance, might be associated with vessels constriction [25]. In line with this finding further mediation analysis of the present study found a significant association between khat chewing during pregnancy and gestational hypertension and gestational hypertension and fetal growth restriction. Normal growth of unborn fetus in the intrauterine life greatly depend on the healthy growth and appropriate attachment of umbilical cord to the placenta [26, 27]. In the present study abnormal cord insertion (marginal), abnormal umbilical cord coiling (both hypo and hyper coiling) and umbilical cord true knots were significantly higher among births of khat chewer cohorts compared to births of non-khat chewer counterparts. Cord abnormalities are highly related with abnormalities in development and function of the placenta [28]. In addition, impaired vascular development in placenta is closely associated with cord abnormalities [29, 30]. As reported in a previous study [31] the peripheral cord insertion compared to central cord insertion was significantly associated with fetal growth restriction. This may be due to the fact that central insertion of cords to the placenta will enables vessels to be stable and hence, will shelter from rotational and pressing forces [32] which will interrupt the blood flow, unlike with that of peripheral insertions. In addition, central cord insertions will better enable a sizeable distribution and flow of blood in different placental parts, that will then enable for better growth of the fetus [31]. Previous studies documented both hypo-coiled [32–34] and hyper-coiled [35, 36] umbilical cord being significantly associated with the occurrence of fetal growth restriction. The possible justification could be due to the fact that, hypo-coiling may be associated with solidity of cord and hyper-coiling may lead to rotation of the cord; in both cases may be associated with interfering to fetoplacental blood flow which in turn leads to fetal growth restriction.

The present study established an association between khat chewing during pregnancy and fetal growth restriction, but the association may not be causal. This is the major limitation of the current study. Therefore, in the interpretations of the present finding this limitation must be considered. However, this study has the following strengths. One is being a prospective cohort, as it establishes temporal relationships between khat chewing during pregnancy and fetal growth restriction. In addition, being a prospective cohort, the chance of missing data will be significantly reduced. The other strength is that the present study showed the mediation analysis model, thereby explaining the mechanism through which khat chewing during pregnancy can influence fetal growth restriction. At last, up to our effort, the present study is primary in its nature, especially in demonstrating the effect of khat chewing during pregnancy on fetal growth restriction in a prospective cohort study design approach in Ethiopia.

## Conclusions

In sum, the present study showed khat chewing during pregnancy is not simply affected the mothers, but it also affected the unborn fetuses. Therefore, the health workers as well as the local community and religious leaders should give high emphasis on provision of health education regarding the damage of chewing khat by pregnant mothers, with especial focus of the effects on their fetuses.

## Data Availability

Data will be available upon request of the corresponding author.

## Abbreviations

ARR: Adjusted Relative Risk
ANC: Ante Natal Care
AR: Attributable Risk
BMI: Body Mass Index
CI: Confidence Interval
FGR: Fetal Growth Restriction
GLM: Generalized Linear Model
IQR: Inter Quartile Range
LMP: Last Menstrual Period
MUAC: Mid-Upper Arm Circumference
PROM: Pre-labor Rupture of Membranes
SGA: Small for Gestational Age
SD: Standard deviation
WHO: World Health Organization
